# Myo‐Inositol Deficiency, Structural Brain Changes, and Cerebral Perfusion Alterations in Classic Galactosemia: Preliminary Insights From a Multiparametric MRI Study

**DOI:** 10.1002/jimd.70097

**Published:** 2025-10-13

**Authors:** Eva Niess, Fabian Niess, Wolfgang Bogner, Alena Svatkova, Marion Herle, Lisa Laußner, Lukas Hingerl, Bernhard Strasser, Maximilian Pichler, Vassiliki Konstantopoulou, Miriam Hufgard‐Leitner, Dominic Buchinger, Ivan Milenkovic, Alexandra Kautzky‐Willer, Thomas Stulnig, Thomas Scherer

**Affiliations:** ^1^ High Field MR Center, Department of Biomedical Imaging and Image‐Guided Therapy Medical University of Vienna Vienna Austria; ^2^ Christian Doppler Laboratory for MR Imaging Biomarkers (BIOMAK), Department of Biomedical Imaging and Image‐Guided Therapy Medical University of Vienna Vienna Austria; ^3^ Danish Research Centre for Magnetic Resonance, Department of Radiology and Nuclear Medicine Copenhagen University Hospital – Amager and Hvidovre Copenhagen Denmark; ^4^ Department of Radiology and Nuclear Medicine Copenhagen University Hospital – Amager and Hvidovre Copenhagen Denmark; ^5^ Division of Pediatric Pulmonology, Allergology and Endocrinology, Austrian Newborn Screening, Department of Paediatrics and Adolescent Medicine Medical University of Vienna Vienna Austria; ^6^ Division of Endocrinology and Metabolism, Department of Medicine III Medical University of Vienna Vienna Austria; ^7^ Department of Paediatrics and Adolescent Medicine Medical University of Vienna Vienna Austria; ^8^ Department of Neurology Medical University of Vienna Vienna Austria; ^9^ Department of Medicine III and Karl Landsteiner Institute for Metabolic Diseases and Nephrology Klinik Hietzing Vienna Austria

**Keywords:** cerebral blood flow, classic galactosemia, cortical thickness, MR spectroscopic imaging, MR spectroscopy, myo‐inositol

## Abstract

Classic galactosemia is a rare metabolic disorder resulting from galactose‐1‐phosphate uridylyltransferase deficiency, which disrupts normal galactose metabolism, leading to toxic accumulation of galactose‐1‐phosphate and galactitol. Despite early dietary intervention, patients remain at risk for long‐term neurological impairments, including cognitive deficits, motor speech disorders, and psychiatric conditions. The mechanisms driving these persistent abnormalities remain unclear. This study investigated brain metabolic and structural alterations in adults with classic galactosemia using advanced multiparametric MRI. Six patients (3 males, 3 females; mean age 34.0 ± 7.3 years) adhering to lifelong galactose‐restricted diets and six age‐ and sex‐matched controls underwent 3T and 7T MRI, including T1‐weighted imaging, pseudo‐continuous arterial spin labeling, and high‐resolution MR spectroscopic imaging. Patients exhibited significantly lower myo‐inositol (mIns) concentrations in cerebellum (*p* = 0.007), putamen (*p* = 0.023), and cerebral white matter (*p* = 0.001), reflecting a chronic mIns deficiency despite dietary management. Structural analyses revealed reduced volumes of white matter (*p* < 0.001), bilateral putamen (*p* < 0.038), and left thalamus (*p* = 0.044); alongside increased cortical thickness and reduced cortical surface area, indicating abnormal cortical maturation, particularly in regions associated with motor and cognitive processing. Additionally, cerebral blood flow was elevated in emotion‐processing regions, including bilateral amygdala (*p* < 0.022) and thalamus (*p* < 0.038). These preliminary findings highlight persistent neurological alterations in classic galactosemia despite dietary management and suggest that chronic mIns deficiency may contribute to the pathophysiology. They underscore the need for larger, longitudinal studies to confirm these results, investigate potential correlations with clinical severity and biochemical markers, and explore therapeutic strategies aimed at modulating mIns metabolism.

## 1 Introduction

Classic galactosemia is a rare inborn error of metabolism caused by mutations in the galactose‐1‐phosphate uridylyltransferase (GALT) gene. This enzyme deficiency disrupts normal galactose metabolism, leading to the toxic accumulation of galactose and its derivatives—galactose‐1‐phosphate (G‐1‐P) and galactitol—in red blood cells and many other cells, tissues, and body fluids, such as urine and plasma [1]. Early detection via newborn screening, followed by immediate dietary intervention with a galactose‐restricted (i.e., lactose‐free) diet, effectively prevents severe neonatal complications, including liver failure, sepsis, cataract, and death [2, 3]. However, despite early and lifelong dietary treatment, patients remain at risk for significant long‐term complications, particularly central nervous system (CNS)‐related neurodevelopmental delay [4] or premature ovarian failure in females [5].

The most prevalent and debilitating CNS‐related manifestations include cognitive impairments, learning difficulties, motor speech and language deficits (e.g., childhood apraxia of speech, dysarthria), movement disorders (e.g., tremor, ataxia), and psychiatric conditions such as depression and anxiety, emerging regardless of treatment timing and despite adherence to a strict diet [6, 7, 8, 9, 10]. These persistent neurological and neuropsychological deficits suggest an underlying brain pathology that remains insufficiently understood and inadequately addressed by current treatment strategies. Given the primary involvement of the CNS, multiple neuroimaging studies have sought to characterize the structural and functional consequences of classic galactosemia. Findings in both adolescents and adults reveal widespread abnormalities, including white matter (WM) microstructure pathology [11], altered gray matter (GM) density [12], disrupted functional networks [13, 14], and cerebral and cerebellar atrophy [15, 16]. Additionally, metabolic brain alterations have been suggested by a positron emission tomography study demonstrating abnormal glucose metabolism across multiple brain regions [17]. These findings indicate that classic galactosemia affects brain structure and function on multiple levels, yet the precise mechanisms driving these abnormalities remain unclear.

A key hypothesis in the pathophysiology of galactosemia involves disrupted myo‐inositol (mIns) homeostasis. MR spectroscopy (MRS) studies in untreated newborns with galactosemia detected a galactitol peak and up to a 60% reduction in the myo‐inositol‐to‐total creatine (mIns/tCr) ratio, which normalized within weeks of dietary galactose restriction [18, 19]. This mIns deficiency is attributed to the toxic accumulation of G‐1‐P and galactitol, which inhibit inositol monophosphatase (IMPA‐1), preventing mIns regeneration, and suppress sodium‐myoinositol‐contransporter‐1 (SMIT‐1), thereby reducing mIns uptake into brain cells [20]. Additionally, galactitol‐induced osmotic imbalance further depletes mIns, which also functions as an osmolyte.

mIns deficiency is likely to contribute to galactosemia‐related brain pathology via several mechanisms: disruption of signal transduction (e.g., protein kinase C, PI3K/Akt/mTOR), dysregulated Ca2+ signaling, or altered osmotic regulation [3]. At the biochemical level, mIns is converted into phosphatidylinositol in the endoplasmic reticulum membrane and further transported at the plasma membrane, where it is phosphorylated to form phosphoinositides, which regulate diverse cellular processes including endosomal vesicle trafficking, autophagy, cytoskeletal reorganization, lipid homeostasis, ion channel activity, Ca2+, and protein kinase C signaling [21]. mIns depletion may therefore impair these pathways, with direct effects on neurons and glia, ultimately contributing to neuronal abnormalities in classic galactosemia. Recent studies in galactosemia mouse models suggest that supplementation with mIns showed a positive effect on gonadal and brain pathology [22] as well as on motor and behavioral outcomes [23].

While a dietary intervention normalizes mIns levels in newborns with galactosemia [19], endogenous galactose production persists throughout life, raising the possibility that chronic subclinical mIns deficiencies contribute to the sustained neurological, cognitive, and ovarian dysfunction observed in treated adults [24]. However, prior MRS studies in diet‐treated adults have reported no significant abnormalities in mIns or other brain metabolites [25, 26]. Recent advancements in MRS methodology, including short‐echo or echo‐less MR spectroscopic imaging (MRSI) [27], and the increased sensitivity of high‐field 7T MRI, now provide improved detection of J‐coupled metabolites like mIns, allowing for a more precise evaluation of metabolic disturbances in classic galactosemia.

In this study, we aim to investigate the presence of persistent mIns deficiency in diet‐treated galactosemia patients using state‐of‐the‐art MRSI and MRS techniques. We focus on brain regions known to be affected in this condition, including the cerebrum, putamen, and cerebellum, to assess potential metabolic disruptions. Additionally, we perform a detailed analysis of cortical and subcortical GM, incorporating measures of cortical thickness, volume, surface area, and cerebral perfusion—parameters that have not been systematically explored in previous studies. By providing a more comprehensive characterization of structural and metabolic alterations in galactosemia, our study aims to further clarify unresolved questions regarding disease pathophysiology.

## 2 Methods

This study was approved by the ethics committee of the Medical University of Vienna (EK #1817/2015). Written informed consent was obtained from all participants.

### 2.1 Participants

Six patients with classic galactosemia (3 males, 3 females; mean age ± SD: 34.0 years ±7.3) and six age‐ and sex‐matched healthy controls (3 males, 3 females; mean age ± SD: 34.3 years ±7.1) participated in this study. The patients were recruited from the outpatient clinic for inherited metabolic diseases in adulthood, Division of Endocrinology and Metabolism at the Department of Medicine III, Medical University of Vienna. Inclusion criteria included a confirmed diagnosis of classic galactosemia via newborn screening through GALT enzyme activity assay and/or GALT gene mutational analysis, adherence to a galactose‐restricted diet since diagnosis, and an age of at least 18 years. Exclusion criteria included the presence of secondary neurological or metabolic disorders that could influence clinical outcomes, as well as contraindications to MRI. One patient was excluded from the 7T MRI examination due to the presence of an implant. Patient characteristics are detailed in Table 1.

**TABLE 1:** Clinical findings for patients diagnosed with galactosemia.

ID	Age (y)	Sex	BMI (kg/m^2^)	GALT^a^ (U/gHb)	G‐1‐P^b^ (mg/dL)	GAL^c^ (mg/dL)	GALT genotype	Other conditions	Medication
G1	32	F	20	1.22	4.67	3.21	p.Gln188Arg/p.Gln188Arg	Osteopenia, primary amenorrhea due to primary ovarian insufficiency	Hormone replacement therapy, calcium, vitamin D
G2	30	F	21	1.19	4.83	2.91	p.Gln188Arg/p.Gln188Arg	Primary ovarian insufficiency	Calcium, vitamin D
G3	42	M	25	n/a	3.57	3.8	p.Gln188Arg/p.Lys285Asn	Underwent surgery for acoustic neuroma, postoperative partial facial palsy, osteopenia, neurogenic detrusor overactivity	Calcium, vitamin D, tolterodine
G4	42	M	20	2.3	3.45	2.7	unknown	Developmental delay, epilepsy	Levetiracetam
G5	23	F	13	0	3.46	2.7	p.Met129Thr/p.Trp154Ter	Anorexia nervosa, osteoporosis, thalassemia trait, primary ovarian insufficiency	Hormone replacement therapy, calcium, vitamin D
G6	35	M	20	n/a	3.57	3.2	p.Asp28Tyr/p.Gln38Pro	Asthma	Calcium, vitamin D, Fluticasone/Salmeterol

^a^ GALT enzyme activity—reported in units per gram of hemoglobin.

^b^ G‐1‐P—galactose‐1‐phosphate concentration in erythrocytes.

^c^ GAL—galactose concentration in erythrocytes.

### 2.2 Neuropsychological Assessment

Four of six patients with galactosemia underwent a battery of neuropsychological tests that included the German Version of the Auditory Verbal Learning Test (VLMT) [28], the Beery‐Buktenica Developmental Test of Visual‐Motor Integration (Beery VMI; Sixth Edition) [29], and the Test of Attentional Performance (TAP; version 2.0) [30], in particular subtests Alertness, Incompatibility, Divided attention, and Go/Nogo.

### 2.3 MRI Acquisition

Data were acquired at a 3.0‐T Siemens Prisma whole‐body scanner and a 7.0‐T Siemens Magnetom whole‐body scanner (both Siemens Healthineers, Erlangen, Germany) equipped with a 64‐channel head coil (Siemens Healthineers, Erlangen, Germany) and a 32‐channel head coil (Nova Medical, Wilmington, MA, US), respectively.

The following sequences were included in the 3T MRI protocol:

- T1‐weighted imaging: Magnetization Prepared Rapid Gradient Echo (MPRAGE) sequence with 0.8 mm isotropic voxel resolution, TE/TI/TR = 2.24/1060/2400 ms, 208 slices, GRAPPA factor 2, and a total acquisition time (TA) of 6:38 min.
- T2‐weighted imaging: Fluid Attenuated Inversion Recovery (FLAIR) sequence with 1 mm isotropic voxel resolution, TE/TI/TR = 414/1800/5000 ms, 160 slices, GRAPPA factor 3, and TA = 5:12 min.
- Arterial spin labeling (ASL): Pseudo‐continuous ASL (pCASL) sequence with a multi‐band echo planar imaging (EPI) readout [31], spatial resolution of 2.5 × 2.5 × 2.3 mm^3^, TE/TR = 19/3700 ms, 60 slices, multiband factor 6, single post‐labeling delay, labeling duration/post‐labeling delay = 1600/1600 ms, 43 label/control pairs, and TA = 5:39 min. A calibration image (M0) was also acquired with TR = 8000 ms for quantification purposes.

The following sequences were included in the 7T MRI protocol:

- T1‐weighted imaging: Magnetization Prepared 2 Rapid Gradient Echoes (MP2RAGE) sequence with 0.75 mm isotropic voxel resolution, TE/TI_1_/TI_2_/TR = 4.12/700/2700/4000 ms, 224 slices, GRAPPA factor 3, and TA = 6:02 min.
- MR Spectroscopic Imaging (MRSI): 3D‐echo‐less MRSI sequence with concentric ring readout [32], spatial resolution of 5.0 × 5.0 × 4.3 mm^3^, matrix size of 44 × 44 × 31, TE/TR = 1.3/320 ms, flip angle of 32°, spectral bandwidth of 2778 Hz, three temporal interleaves, WET water suppression, and TA = 4:58 min. The volume of interest (VOI) was aligned with anterior commissure—posterior commissure line, covering cerebrum from the vertex to the inferior border of lateral ventricles.
- Single voxel spectroscopy: semi‐LASER sequence with excellent reproducibility [33, 34] provided by the University of Minnesota under a C2P agreement with TE/TR = 28/8600 ms, spectral bandwidth of 6000 Hz, 2048 complex points, VAPOR water suppression, and FASTMAP B_0_ shimming [35] within two VOI:
  ○ Left putamen with voxel volume of 35 × 10 × 15 mm^3^, TA = 10:36 min, and 64 averages.
  ○ Cerebellum (placed symmetrically about the midline and primarily covering the cerebellar vermis) with volume of 25 × 10 × 25 mm^3^, TA = 11:38 min, and 71 averages. Reproducible voxel placements were based on anatomical landmarks. During both single voxel acquisitions four unsuppressed water spectra were acquired as a reference to derive metabolite concentration estimates. Additional sequence parameters are listed in Table S1 following the Minimum Reporting Standards for MR Spectroscopy (MRSinMRS) experts’ consensus recommendations [36].

### 2.4 MRI Processing Analysis

The presence of WM hyperintensities was evaluated using T2‐weighted FLAIR images. Volumetric segmentation and cortical reconstruction of T1‐weighted MPRAGE images were performed using the automated FreeSurfer *recon‐all* pipeline (v7.4.1; https://surfer.nmr.mgh.harvard.edu/). Cortical parcellation was based on the Desikan‐Killiany atlas, resulting in 62 cortical regions of interest (ROIs), with 31 ROIs for each hemisphere. For each ROI, cortical thickness, surface area, and volume were estimated and included in the analyses. Additionally, the results of volumetric segmentation were analyzed.

Pseudo‐continuous ASL data were pre‐processed and quantified using FSL's BASIL toolbox [37]. Pre‐processing steps included image realignment to correct for motion artefacts, distortion correction using a fieldmap, and pairwise subtraction of label and control images to generate perfusion‐weighted images, which were subsequently averaged. Brain extraction was performed using FSL BET, and quantitative cerebral blood flow (CBF) maps were calculated in units of milliliters of blood per 100 g of tissue per minute (ml/100 g/min). CBF quantification employed a single‐compartment kinetic model and voxel‐wise calibration using the M0 image. The resulting CBF maps were co‐registered with T1‐weighted MRI images and aligned with the cortical parcellation and volumetric segmentation outputs from FreeSurfer. Average regional CBF values were then calculated for each ROI based on the Desikan‐Killiany atlas obtained in the previous step.

3D‐MRSI data were processed with an in‐house developed pipeline. Shortly, this included iMUSICAL coil combination [38], k‐space reconstruction using non‐Cartesian discrete Fourier transform, spatial Hamming filtering, channel‐wise noise decorrelation, and lipid signal removal using L2‐regularization [39]. Spectral fitting was performed in LCModel (version 6.3; http://s‐provencher.com/lcmodel.shtml) with a simulated basis set that included 17 brain metabolites and a measured macromolecular background [40] over an evaluation range of 1.8–4.2 ppm. Averaged metabolic ratios were calculated for two ROIs: cerebral white matter (including voxels with a minimum content of 80% WM) and cortical gray matter (voxels with a minimum content of 50% GM).

Individual spectra acquired with semi‐LASER sequence were corrected for small frequency and phase fluctuations and subsequently averaged. Averaged spectra were analyzed with LCModel (v6.3; http://s‐provencher.com/lcmodel.shtml) in the frequency range 0.5–4.2 ppm utilizing the unsuppressed water spectrum as an internal reference. Metabolite concentrations in units of molarity (mM) were determined after correcting for tissue water and tissue composition (GM, WM) using FSL‐MRS, alongside relaxation correction based on literature values. The preprocessing, analysis, and quantification followed experts' consensus recommendations [41].

### 2.5 Statistical Analysis

All statistical analyses were performed using SPSS (version 28, SPSS Inc., Chicago, Illinois, USA). A one‐way analysis of covariance (ANCOVA) was conducted to examine differences in cortical thickness, surface area, and volumes between study groups, with estimated total intracranial volume (eTIV) included as a covariate to account for variations in participant's head sizes. Differences in cerebral blood flow and metabolite concentrations and ratios between study groups were assessed using independent‐samples t‐tests. Correlations between MRI metrics and results of neuropsychological testing were analyzed using the Pearson correlation coefficient or partial correlation controlling for eTIV, respectively.

All tests were two‐tailed, with a significance threshold set at *p* < 0.05. To account for potential bias from multiple comparisons arising from testing multiple ROIs, *p* values of regional cross‐sectional analyses were adjusted using the false discovery rate (FDR) correction method (i.e., Benjamini‐Hochberg). Results of correlation analyses were not FDR‐adjusted due to the limited number of subjects.

## 3 Results

### 3.1 Metabolite Concentrations and Ratios

**FIGURE 1:**
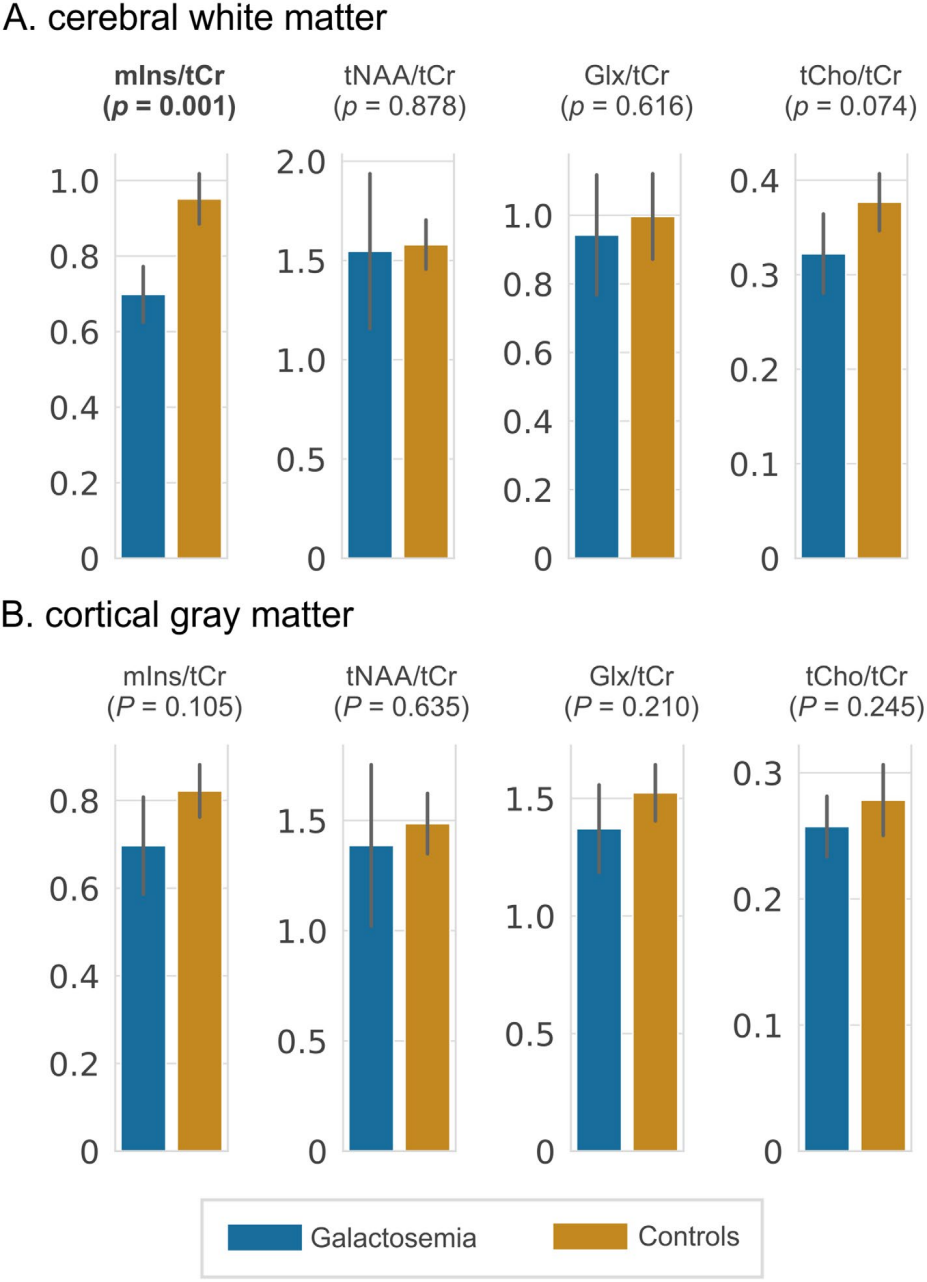
Mean metabolic ratios of myo‐inositol (mIns), total *N*‐acetylaspartate (tNAA), glutamate + glutamine (Glx), and total choline (tCho) normalized to total creatine (tCr) within A. cerebral white matter and B. cortical gray matter of galactosemia patients and controls. Patients exhibited lower levels of mIns/tCr in cerebral white matter.

**FIGURE 2:**
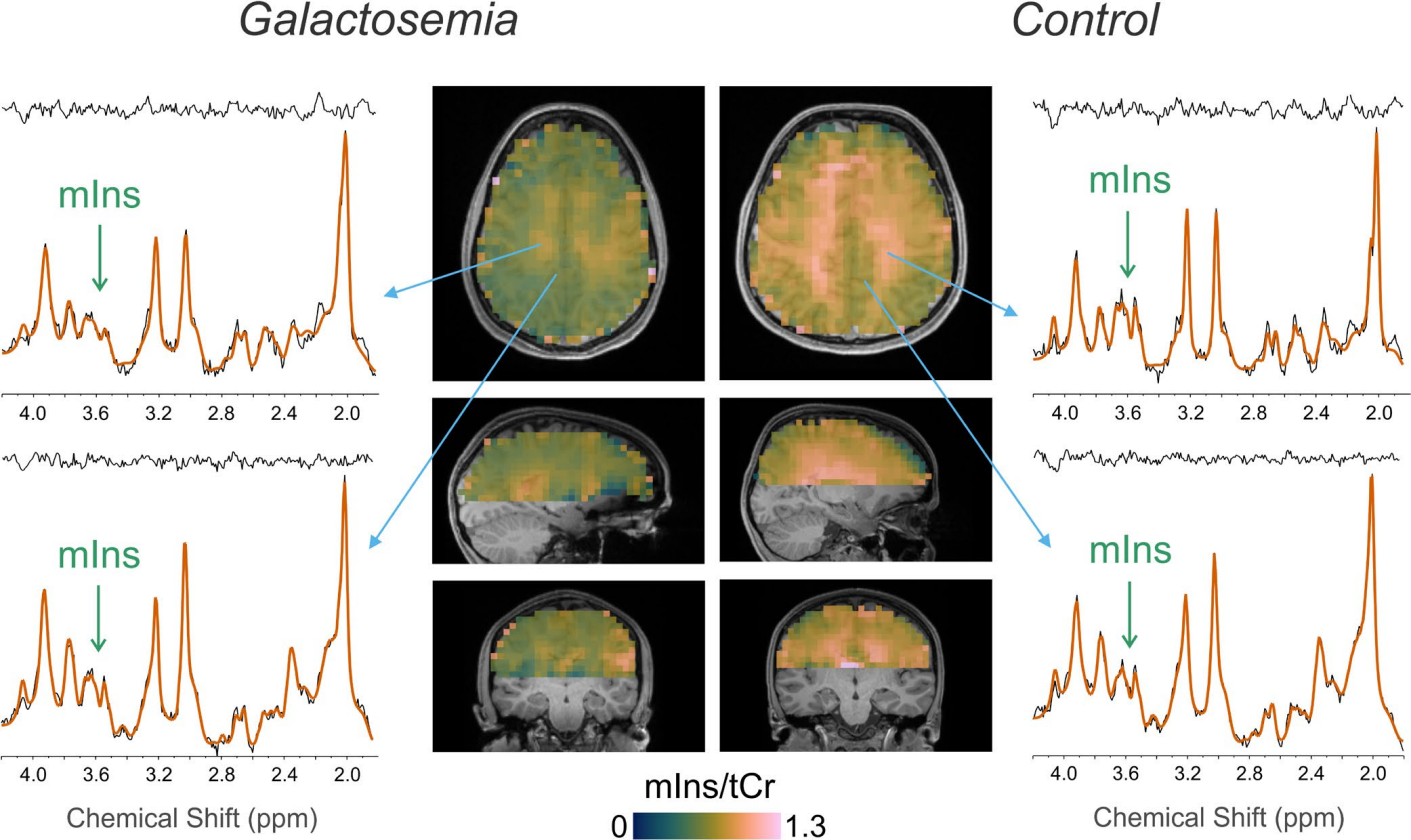
Sample metabolic maps and spectra acquired with 3D‐MR spectroscopic imaging in galactosemia patient and control. Widespread reduction of myo‐inositol (mIns) normalized to total creatine was detected in patients, particularly within their white matter.

**FIGURE 3:**
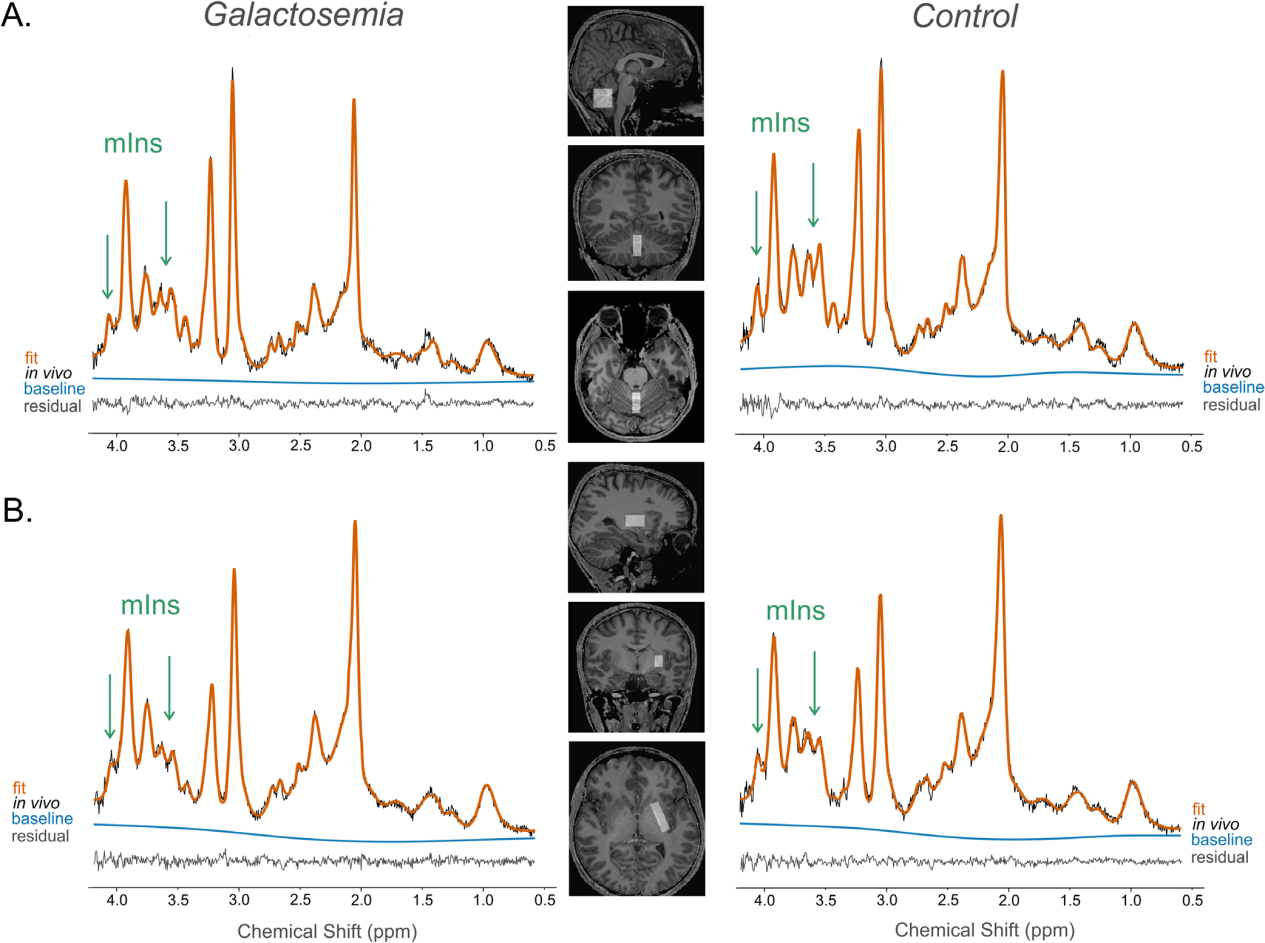
Sample spectra acquired with semi‐LASER from A. the cerebellum and B. left putamen of galactosemia patients and control. Patients exhibited lower myo‐inositol (mIns) concentrations in both regions.

In cerebral WM, galactosemia patients exhibited a lower ratio of myo‐inositol to total creatine (mIns/tCr) (*p* = 0.001, mean difference [MD] ± standard error [SE]: −0.25 ± 0.05). No differences in mIns/tCr were observed in cortical GM (*p* = 0.105) and for other metabolic ratios (Figure 1). Sample spectra and metabolic maps from 3D‐MRSI are presented in Figure 2. Sample spectra from the cerebellum and left putamen are shown in Figure 3. Patients with galactosemia exhibited significantly lower myo‐inositol (mIns) concentrations in both VOI (cerebellum: *p* = 0.007, MD ± SE: −0.92 mM ± 0.27; putamen: *p* = 0.023, MD ± SE: −1.08 mM ± 0.58). Additionally, *N*‐acetylaspartylglutamate (NAAG) concentrations in the cerebellum were lower in galactosemia patients (*p* = 0.021, MD ± SE: −0.24 mM ± 0.09) (Table 2). To facilitate the comparison with studies conducted at lower field strength, we additionally report mIns/tCr ratios (uncorrected, output of LCModel) from both VOI in Table 2. Consistent with our main findings, patients showed lower mIns/tCr ratios compared to healthy controls in both regions: cerebellum (*p* = 0.014, MD ± SE: −0.12 ± 0.04) and putamen (*p* = 0.021, MD ± SE: −0.12 ± 0.04).

**TABLE 2:** Mean metabolite concentrations and Cramér‐Rao lower bounds measured in the cerebellum and left putamen of galactosemia patients and healthy controls.

Cerebellum										
Metabolite	Galactosemia				Controls				*t*	*p*
	Conc.	SD	CRLB	SD	Conc.	SD	CRLB	SD		
	(mM)	(mM)	(%)	(%)	(mM)	(mM)	(%)	(%)		
GABA	1.44	0.25	19.2	4.5	1.56	0.29	18.5	6.3		0.523
Gln	3.06	0.47	7.8	1.8	3.33	0.56	7.5	1.5		0.464
Glu	8.26	0.68	2.8	0.7	8.75	0.54	2.7	0.5		0.256
GSH	1.58	0.24	9.0	2.3	1.67	0.31	8.3	2.1		0.623
mIns	6.42	0.47	2.4	0.5	7.34	0.34	1.8	0.4	−3.44	0.007
NAA	8.62	0.58	1.8	0.4	8.90	0.92	1.7	0.5		0.625
NAAG	0.98	0.17	11.4	3.9	1.21	0.07	8.2	1.9	−2.79	0.021
sIns	0.30	0.12	15.6	4.7	0.28	0.11	17.2	6.0		0.78
Tau	2.85	0.23	7.4	1.0	3.21	0.40	6.0	0.6		0.142
tCho	2.20	0.21	2.4	0.5	2.40	0.30	1.8	0.4		0.325
tCr	10.79	0.45	1.0	0	10.86	0.63	1.0	0		0.844
mIns/tCr	0.58	0.09	—	—	0.70	0.04	—	—	−2.84	0.014
Fractional tissue composition [%]										
CSF	0.15 ± 0.03				0.15 ± 0.06					
GM	0.58 ± 0.01				0.61 ± 0.03					
WM	0.27 ± 0.03				0.24 ± 0.04					

Putamen										
Metabolite	Galactosemia				Controls				*t*	*p*
	Conc.	SD	CRLB	SD	Conc.	SD	CRLB	SD		
	(mM)	(mM)	(%)	(%)	(mM)	(mM)	(%)	(%)		
GABA	1.50	0.11	20.4	3.0	1.46	0.15	29.0	3.0		0.813
Gln	3.22	0.3	8.4	0.8	3.21	0.49	11.8	2.0		0.984
Glu	7.97	0.58	3.2	0.4	8.44	0.32	3.8	0.7		0.16
GSH	1.05	0.24	14.8	2.5	1.43	0.42	13.0	2.1		0.139
mIns	4.12	0.47	3.8	0.7	5.23	0.68	3.6	0.5	−2.73	0.023
NAA	8.50	0.26	2.0	0	8.59	0.25	2.6	0.5		0.634
NAAG	1.02	0.20	13.6	5.4	1.12	0.36	17.3	10.5		0.615
sIns	0.29	0.10	24.4	8.6	0.39	0.09	18.4	5.0		0.174
Tau	1.49	0.53	16	8.6	1.60	0.36	15.0	4.1		0.735
tCho	1.39	0.19	3.6	0.5	1.63	0.33	3.7	0.9		0.233
tCr	9.17	0.2	1.4	0.5	8.58	0.58	1.7	0.5		0.08
mIns/tCr	0.50	0.09	—	—	0.62	0.06	—	—	−2.79	0.021
Fractional tissue composition [%]										
CSF	0.01 ± 0.01				0.01 ± 0.01					
GM	0.43 ± 0.10				0.45 ± 0.07					
WM	0.56 ± 0.11				0.54 ± 0.05					

Abbreviations: CSF: cerebrospinal fluid; GABA: gamma‐aminobutyric acid; Gln: glutamine; Glu: glutamate; GM: gray matter; GSH: glutathione; mIns: myo‐inositol; NAA: *N*‐acetylaspartylglutamate; NAAG: *N*‐acetylaspartylglutamate; sIns: scyllo‐inositol; Tau: taurine; tCho: total choline; tCr: total creatine; WM: white matter.

### 3.2 Volumetry

Patients exhibited significantly lower bilateral cerebral white matter (WM) volumes compared to controls (left hemisphere [LH]: *p*_corr_ < 0.001, estimated mean difference [eMD] ± standard error [SE]: −63 405 mm^3^ ± 12 921; right hemisphere [RH]: *p*_corr_ < 0.001, eMD ± SE: −59 975 mm^3^ ± 12 312). Subcortical gray matter (GM) volume was also reduced in patients (*p*_corr_ = 0.039, eMD ± SE: −6440 mm^3^ ± 2024). Within the subcortical GM, reductions were observed in the bilateral putamen (left: *p*_corr_ = 0.035, eMD ± SE: −1080 mm^3^ ± 294; right: *p*_corr_ = 0.038, eMD ± SE: −1161 mm^3^ ± 351) and the left thalamus (*p* = 0.044, eMD ± SE: −1003 mm^3^ ± 336). Bilateral lateral ventricles were larger in patients compared to controls (left: eMD ± SE: +13 966 mm^3^ ± 5099; right: eMD ± SE: 13 159 mm^3^ ± 4824); however, this difference did not remain significant after FDR correction (both *p*_*corr*_ = 0.058). The results are summarized in Table 3.

**TABLE 3:** Results of the volumetric analysis.

	*F*	df	*p*	*p*_corr_	*η*_p_^2^	eMD [mm^3^]	SE
LH Cerebral WM Volume	24.081	1	< 0.001	< 0.001	0.728	−63 405	12 921
RH Cerebral WM Volume	23.731	1	< 0.001	< 0.001	0.725	−59 975	12 312
LH Cortex Volume	1.160	1	0.31	0.369	0.114	−9061	8415
RH Cortex Volume	0.842	1	0.383	0.435	0.086	−7221	7870
Subcortical GM Volume	10.127	1	0.011	0.039	0.529	−6440	2024
Left lateral ventricle	7.502	1	0.023	0.058	0.455	13 966	5099
Right lateral ventricle	7.440	1	0.023	0.058	0.453	13 159	4824
Left cerebellum WM	1.989	1	0.192	0.320	0.181	−2210	1567
Left cerebellum cortex	1.316	1	0.281	0.369	0.128	−5244	4572
Right cerebellum WM	2.136	1	0.178	0.318	0.192	−2108	1442
Right cerebellum cortex	1.247	1	0.293	0.369	0.122	−5053	4526
Left thalamus	9.142	1	0.014	0.044	0.504	−1003	336
Right thalamus	4.134	1	0.073	0.152	0.315	−579	285
Left putamen	11.980	1	0.007	0.035	0.571	−1018	294
Right putamen	10.939	1	0.009	0.038	0.549	−1161	351
Left caudate	0.061	1	0.811	0.845	0.007	−60	243
Right caudate	0.004	1	0.949	0.949	0.000	−20	303
Left pallidum	1.536	1	0.247	0.369	0.146	−146	118
Right pallidum	0.644	1	0.443	0.482	0.067	−116	145
Left hippocampus	1.194	1	0.303	0.369	0.117	−321	294
Right hippocampus	4.316	1	0.068	0.152	0.324	−622	300
Left amygdala	1.504	1	0.251	0.369	0.143	−170	139
Right amygdala	3.870	1	0.081	0.156	0.301	−342	174

Abbreviations: *η*_p_^2^: partial eta‐squared (= estimate of effect size); eMD: estimated mean difference; GM: gray matter; LH: left hemisphere; *p*_corr_: false discovery rate (FDR)‐adjusted *p*; RH: right hemisphere; SE: standard error; WM: white matter.

### 3.3 Cortical Thickness and Surface Area

No significant differences were observed in total cortical volume or within individual ROIs. However, patients exhibited bilaterally lower mean cortical surface area (LH: *p*_corr_ = 0.003, eMD ± SE: −13838.4 mm^2^ ± 3421.6; RH: *p*_corr_ = 0.004, eMD ± SE: −13160.5 mm^2^ ± 3500.2) and higher mean cortical thickness (LH: *p*_corr_ = 0.008, eMD ± SE: 0.175 mm ± 0.052; RH: *p*_corr_ = 0.002, eMD ± SE: 0.176 mm ± 0.042) compared to controls.

**FIGURE 4:**
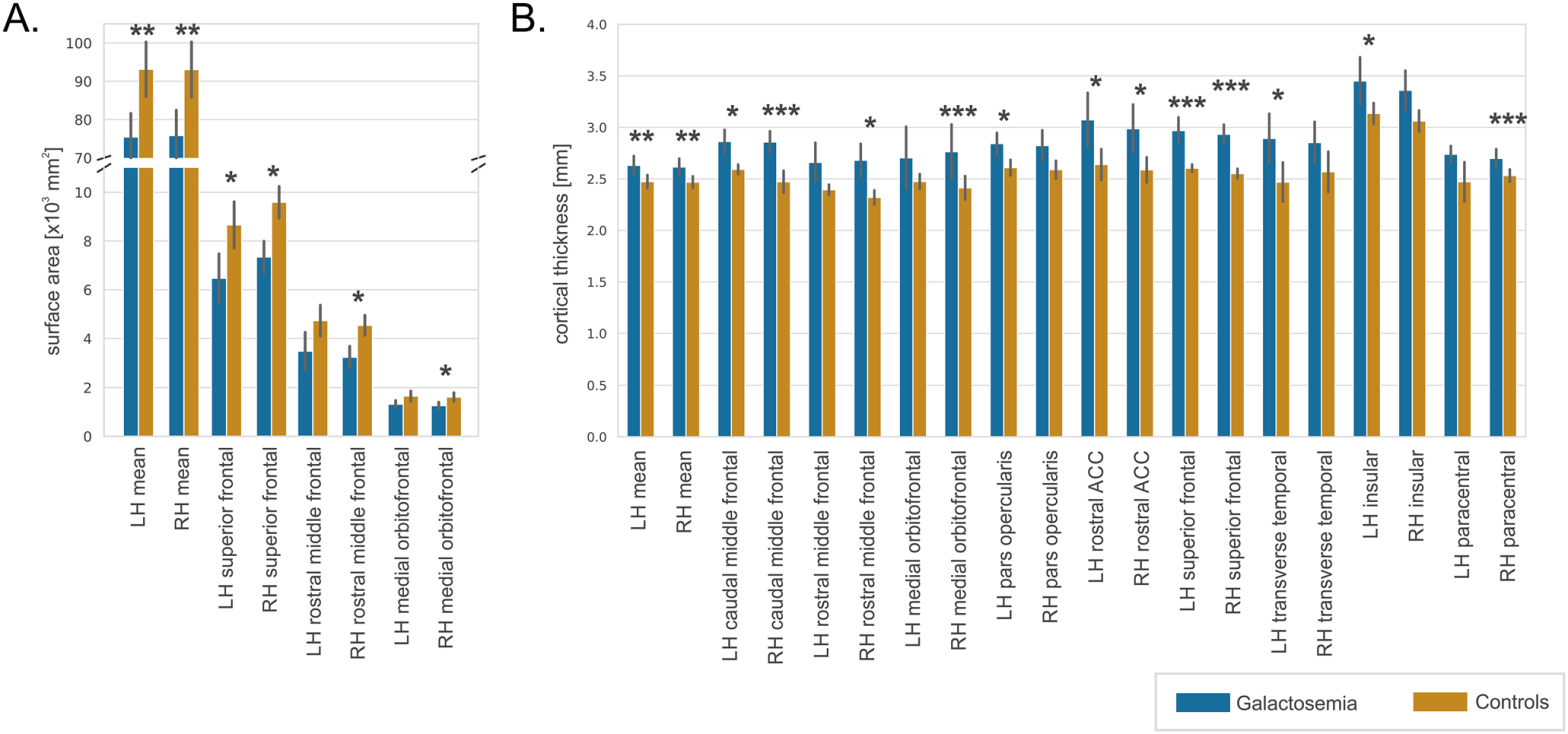
Results from ROI analysis of A. surface area and B. cortical thickness. Patients with galactosemia show lower surface area and increased cortical thickness, bilaterally. The figure shows brain regions where significant (*p_corr_ < 0.05, **p_corr_ < 0.01, ***p_corr_ < 0.001) differences in patients with galactosemia compared to controls were detected in at least one hemisphere (“LH” = left hemisphere, “RH” = right hemisphere). Error bars denote standard deviation. Results from regions without significant differences can be found in Tables S2 and S3.

At the regional level, we observed reduced surface area of the bilateral superior frontal gyrus (LH: *p*_corr_ = 0.016, eMD ± SE: −1644.3 mm^2^ ± 512.0; RH: *p*_corr_ = 0.016, eMD ± SE: −1985.3 mm^2^ ± 389.4), right medial orbitofrontal gyrus (*p*_corr_ = 0.016, eMD ± SE: −225.2 mm^2^ ± 47.1), and right rostral middle frontal gyrus (*p*_corr_ = 0.016, eMD ± SE: −1134.0 mm^2^ ± 259.6) (Figure 4A, Table S2).

In contrast, regional analysis revealed higher cortical thickness of the bilateral superior frontal gyrus (LH: *p*_corr_ < 0.001, eMD ± SE: 0.337 mm ± 0.061; RH: *p*_corr_ < 0.001, eMD ± SE: 0.371 mm ± 0.049), bilateral caudal middle frontal gyrus (LH: *p*_corr_ = 0.016, eMD ± SE: 0.271 mm ± 0.059; RH: *p*_corr_ < 0.001, eMD ± SE: 0.347 mm ± 0.066), bilateral rostral anterior cingulate gyrus (LH: *p*_corr_ = 0.021, eMD ± SE: 0.535 mm ± 0.082; RH: *p*_corr_ = 0.021, eMD ± SE: 0.458 mm ± 0.119), left pars opercularis (*p*_corr_ = 0.025, eMD ± SE: 0.238 mm ± 0.061), left transverse temporal gyrus (*p*_corr_ = 0.036, eMD ± SE: 0.482 mm ± 0.14), left insular gyrus (*p*_corr_ = 0.025, eMD ± SE: 0.397 mm ± 0.103), right medial orbitofrontal gyrus (*p*_corr_ < 0.001, eMD ± SE: 0.483 mm ± 0.095), right paracentral gyrus (*p*_corr_ < 0.001, eMD ± SE: 0.205 mm ± 0.042), and right rostral middle frontal gyrus (*p*_corr_ = 0.012, eMD ± SE: 0.353 mm ± 0.083) (Figure 4B, Table S3).

### 3.4 Cerebral Blood Flow

**FIGURE 5:**
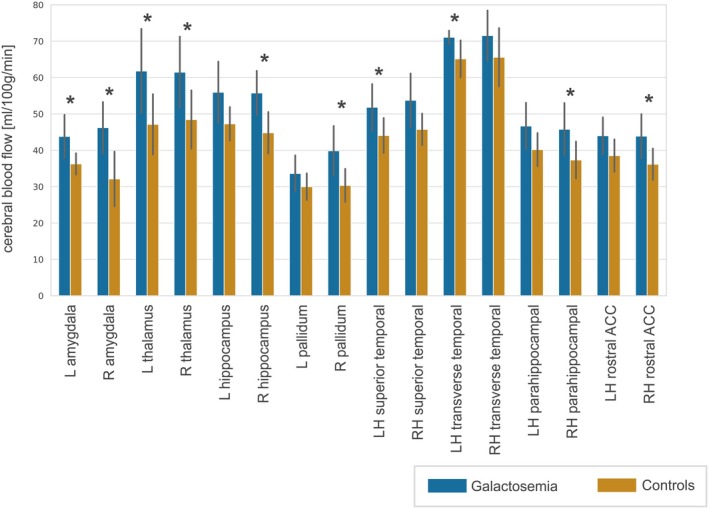
Results from ROI analysis of cerebral blood flow. The figure shows brain regions where significantly higher cerebral blood flow (*p_corr_ < 0.05, **p_corr_ < 0.01, ***p_corr_ < 0.001) in patients with galactosemia compared to controls was detected in at least one hemisphere (“LH” = left hemisphere, “RH” = right hemisphere). Error bars denote standard deviation. Results from regions without significant differences can be found in Table S4.

There were no differences in CBF on the global level, neither within white or gray matter. Regional analysis, however, revealed increased CBF within bilateral amygdala (left: *p*_corr_ = 0.022, MD ± SE: 7.55 mL/100 g/min ± 2.74; right: *p*_corr_ = 0.011, MD ± SE: 14.12 mL/100 g/min ± 4.42), bilateral thalamus (left: *p*_corr_ = 0.037, MD ± SE: 14.67 mL/100 g/min ± 6.00; right: *p*_corr_ = 0.038, MD ± SE: 13.03 mL/100 g/min ± 5.36), right hippocampus (*p*_corr_ = 0.013, MD ± SE: 10.95 mL/100 g/min ± 3.57), right pallidum (*p*_corr_ = 0.022, MD ± SE: 9.53 mL/100 g/min ± 3.43), left superior temporal gyrus (*p*_corr_ = 0.047, MD ± SE: 7.77 mL/100 g/min ± 3.37), left transverse temporal gyrus (*p*_corr_ = 0.036, MD ± SE: 5.94 mL/100 g/min ± 2.41), right parahippocampal gyrus (*p*_corr_ = 0.049, MD ± SE: 8.44 mL/100 g/min ± 3.71), and right rostral anterior cingulate cortex (*p*_corr_ = 0.036, MD ± SE: 7.72 mL/100 g/min ± 3.13) (Figure 5, Table S4).

### 3.5 Correlations With Neuropsychological Testing Results

Significant partial positive correlations were observed between Beery VMI scores and bilateral cerebellar cortex volumes (left: *r* = 0.996, *p* = 0.046; right: *r* = 0.999, *p* = 0.028) as well as left cerebellar white matter volume (*r* = 0.999, *p* = 0.033). Beery VMI scores were also negatively correlated with cortical thickness in multiple regions, including the bilateral pericalcarine, left lateral occipital, right fusiform, and bilateral isthmus cingulate cortex (all *p* < 0.05).

VLMT results showed positive correlation with cerebellar Ins levels (*r* = 0.999, *p* = 0.023) and negative correlation with right isthmus cingulate cortical thickness (*r* = −0.963, *p* = 0.037). TAP scores revealed significant associations as well. Specifically, alertness subtest correlated with cortical thickness of the right rostral middle frontal, right precentral, and right superior parietal gyrus (all *p* < 0.05). Incompatibility subtest scores were linked to thickness in the left rostral middle frontal, left lateral occipital, and left middle temporal cortex (all *p* < 0.05). Full details of significant correlations can be found in Table S5.

## 4 Discussion

This study examined brain abnormalities in adults with classic galactosemia using quantitative multiparametric MRI. Although based on a small, preliminary cohort of six subjects, we found lower mIns levels in the cerebellum, putamen, and cerebral white matter, providing the first in vivo evidence of mIns deficiency in diet‐treated adults, a finding likely enabled by the high spectral resolution of 7T MRI and the improved reliability of quantifying J‐coupled metabolites using short‐echo and echo‐less MRS techniques. Patients also showed reduced cerebral WM and subcortical GM volume, particularly in the left thalamus and bilateral putamen. While cortical GM volume remained unchanged, patients exhibited increased cortical thickness and reduced surface area, suggesting potential abnormalities in brain maturation or pruning. Supporting this hypothesis, we also observed elevated cerebral blood flow in patients, suggesting higher energy demands in less efficient, immature networks. The affected brain regions align with the neuropsychological complications commonly reported in classic galactosemia, including cognitive impairments, motor speech and language difficulties, and psychiatric symptoms.

Our findings of brain mIns deficiency in adults adhering to dietary treatment is novel and may result from endogenous galactose production, primarily via lysosomal hydrolysis of glycolipids, glycoproteins, and proteoglycans [24]. The rate of de novo galactose synthesis was shown to be elevated in patients with galactosemia compared to controls, and it decreases with age [42, 43]. This process leads to the persistent G‐1‐P and galactitol accumulation despite dietary restriction, ultimately reducing mIns levels. Myo‐inositol deficiency has long been linked to cellular distress in galactosemia [3], with decreased levels previously observed in the brains of untreated infants and deceased patients [44]. As both a precursor for membrane‐bound inositol phospholipids and an organic osmolyte, mIns plays a key role in human physiology, regulating cell volume, calcium and protein kinase C signaling, neurotransmission, AKT/mTOR pathways, insulin sensitivity, and vesicular trafficking [20]. Secondary depletion of mIns is therefore hypothesized to impair neuronal and/or glial function through defects in signaling and vesicle trafficking. Given that brain mIns levels normally peak during gestation and early infancy [45], such early deficits may interfere with neurodevelopmental processes and contribute to the long‐term neurological complications of classic galactosemia. Analogously, endogenous galactose synthesis is detectable as early as the prenatal period, suggesting that some of the damage may occur in utero [46]. In a GALT gene‐trapped mouse model, mIns supplementation increased primordial follicle count, normalized cerebellar GM thickness [22], improved motor function, and reduced anxiety‐like behavior [23]. While mIns is widely used for polycystic ovarian syndrome and fertility, supporting evidence remains inconclusive [47]. Nevertheless, its role in FSH‐mediated pathways regulating granulosa cell maturation and oocyte development suggests that mIns deficiency may contribute to primary ovarian insufficiency in galactosemia, warranting further investigation. To our knowledge, no clinical studies examining mIns supplementation in patients with galactosemia have been published. Also, no planned clinical trials are currently listed in the clinictrials.gov database.

Our volumetric findings align with previous studies, confirming reduced WM and bilateral putamen volume in galactosemia patients [12, 15, 16], while also revealing reduced left thalamic volume. As part of the basal ganglia, the putamen is essential for motor control, including speech articulation. The thalamus serves as a relay station for incoming motor and sensory signals to the cerebral cortex. Reduced putamen and thalamic volume may play a role in the cognitive and motor impairments associated with galactosemia. Additionally, given the left thalamus's involvement in language processing, verbal memory, and executive function [48, 49], its reduced volume may contribute to the motor speech disorders (childhood apraxia of speech and dysarthria) commonly observed in this condition.

Our findings of increased cortical thickness are consistent with previous reports of greater GM density in the medial prefrontal, inferior frontal, and insular cortex, with a more extensive effect in the right hemisphere [12]. Likewise, we observed differences primarily in the prefrontal cortex, including the superior frontal, medial frontal, anterior cingulate, and orbitofrontal gyri bilaterally, with greater effect on the right, but also in the left pars opercularis, transverse temporal, and insular gyrus. Cortical thickening may result from osmotic swelling or abnormal myelination; however, its association with observed reduced surface area is more likely to reflect disrupted cortical maturation due to impaired synaptic pruning and/or aberrant folding (e.g., decreased gyrification), leading to less efficient neural circuits [50]. Supporting this, we observed increased CBF, indicating higher metabolic demands, possibly related to greater synaptic density. This aligns with fMRI findings of additional and more extensive brain recruitment during sentence production in galactosemia [13]. Collectively, these findings suggest abnormal GM maturation, potentially caused by early mIns deficiency, which may impair intracellular signaling and brain development [20].

Significant increases in cerebral blood flow were found particularly in areas involved in emotional processing, memory, and cognition, including the amygdala, hippocampus, thalamus, rostral anterior cingulate cortex, and pallidum. Although CBF was also elevated in the bilateral insula, these findings did not survive FDR correction (left: *p*_corr_ = 0.063, right: *p*_corr_ = 0.057). Dysfunction in key regions, particularly the amygdala, insula, and anterior cingulate cortex, has been associated with anxiety and depression, affecting ~50% and 12% of galactosemia patients, respectively. Moreover, prior research has linked elevated perfusion in affective regions to anxiety disorders [51]. Similarly, our findings further suggest that heightened activity in affective circuitry related to anxiety may drive increased regional metabolism and CBF. Cerebral perfusion was also significantly increased in the left superior temporal gyrus and the left transverse temporal gyrus. The left superior temporal gyrus, particularly Wernicke's area, is essential for language comprehension, phonetic processing, and speech perception. The transverse gyrus contains the primary auditory cortex, responsible for early auditory processing, including pitch, tone, and speech sound discrimination. Dysfunction in these regions may underlie the speech and language difficulties observed in galactosemia. Overall, given the limited sample size and modest *p* values, the observed cerebral blood flow alterations should be considered preliminary and require validation in larger, independent cohorts.

In addition to MRI, galactosemia patients underwent a comprehensive neuropsychological assessment, including the Beery VMI, which assesses visual‐motor integration. Beery VMI scores showed significant positive partial correlations with cerebellar volumes, highlighting the cerebellums critical role in fine motor coordination and sensorimotor integration. Although our study did not find significantly reduced cerebellar volumes, previous works have reported such reductions [15]. In contrast, negative associations were observed between Beery VMI scores and increased cortical thickness in regions critical for visual processing and object recognition, including the pericalcarine cortex (primary visual cortex, V1), left lateral occipital cortex, and right fusiform gyrus. These areas are essential for basic visual perception, high‐order visual processing, and shape identification, indicating that long‐term cortical reorganization and altered neurodevelopment may underlie the visual‐perceptual deficits in galactosemia.

VLMT performance, which assesses verbal learning and memory (including encoding, consolidation, and retrieval of verbal information), was positively correlated with cerebellar mIns levels, supporting growing evidence for the cerebellum's role in higher‐order cognitive functions, such as learning and memory [52], alongside traditionally recognized memory‐related regions like the hippocampus. mIns levels in the cerebellum may therefore serve as a potential biomarker for cognitive impairment in galactosemia, reflecting underlying metabolic and neurochemical disruptions that affect both motor and cognitive pathways. Additionally, poorer performance on the VLMT—particularly in recall after interference and delayed recall—was associated with increased cortical thickness in the right isthmus cingulate. This region serves as a bridge between key memory structures, including the cingulate cortex, parahippocampal gyrus, and hippocampus, and its structural integrity has been linked to effective verbal learning and memory retrieval [16].

Results from the TAP revealed positive partial correlations between intrinsic alertness (measured as median reaction time) and increased cortical thickness in the right rostral middle frontal, right precentral, and right superior parietal gyri, reflecting the dominant role of the right hemisphere in attentional control. These regions are crucial for sustained attention, motor preparation, and sensory integration, respectively. Finally, cognitive flexibility (incompatibility subtest, measured as the number of errors) was positively associated with abnormal cortical thickness in the left rostral middle frontal gyrus, part of the dorsolateral prefrontal cortex, a core region for executive functions like working memory, planning, and decision‐making. It also correlated with the left lateral occipital cortex, critical for visual processing and object recognition, and the left middle temporal gyrus, involved in semantic processing and multisensory integration, further highlighting the complex cortical reorganization in galactosemia.

The main limitation of our study is the small sample size. However, the reported cross‐sectional differences remained statistically significant after correcting for multiple comparisons across regions of interest. Larger cohorts with longitudinal follow‐up will be essential to confirm and extend these findings, for example by exploring correlations between mIns deficits, clinical severity, and biochemical markers. It is further important to note that only four out of six patients underwent neuropsychological testing, limiting the generalizability of the correlation analyses. While these preliminary findings are potentially informative, they should be interpreted with caution and can serve as a basis for guiding future research with larger, more representative samples. Finally, we did not investigate the possibility of galactitol quantification at 7T, although this would be highly relevant. The main challenges include its typically low brain concentrations under dietary controls below the in vivo MRS detection threshold of ~1 mM, as well as the spectral overlap of its doublet at ~3.7 ppm with other more abundant metabolites, including glucose and mIns.

In conclusion, this study provides novel yet preliminary insights into brain abnormalities in adults with classic galactosemia using advanced multiparametric MRI. We identified reduced mIns levels, likely due to endogenous galactose production, which may contribute to persistent neurological impairments. Structural analyses confirmed reduced white matter and subcortical gray matter volumes, particularly in the putamen and thalamus, potentially affecting motor and cognitive functions. Increased cortical thickness and cerebral blood flow suggest disrupted maturation and compensatory metabolic activity. Elevated perfusion in affective and language‐related regions may underlie psychiatric symptoms and speech difficulties, highlighting the need for larger, longitudinal studies to confirm these findings, as well as further research into therapeutic strategies targeting mIns metabolism.

## Author Contributions

**E.N.:** validation, formal analysis, investigation, data curation, writing – original draft, visualization. **F.N.:** software, validation, writing – review and editing. **W.B.:** methodology, resources, writing – review and editing. **A.S.:** conceptualization, investigation, writing – review and editing, funding acquisition. **M.H.:** investigation, data curation, writing – review and editing. **L.L.:** investigation, data curation, writing – review and editing. **L.H.:** software, validation. **B.S.:** software, validation. **M.P.:** data curation, writing – review and editing. **V.K.:** investigation, resources. **M.H.‐L.:** investigation, resources. **D.B.:** investigation, resources. **I.M.:** investigation, resources. **A.K.‐W.:** funding acquisition, supervision, project administration. **T.St.:** conceptualization, writing – review and editing, funding acquisition. **T.Sc.:** conceptualization, methodology, resources, writing – review and editing, supervision, project administration.

## Ethics Statement

The patients were recruited from the outpatient clinic for inherited metabolic diseases in adulthood, Division of Endocrinology and Metabolism at the Department of Medicine III, Medical University of Vienna. This study was approved by the ethics committee of the Medical University of Vienna (EK #1817/2015).

## Consent

Written informed consent was obtained from all participants.

## Conflicts of Interest

The authors declare no conflicts of interest.

## Supporting information


**Table S1:** Minimum Reporting Standards for in vivo MR Spectroscopy (MRSinMRS).
**Table S2:** Results of the ROI analysis of cortical surface area.
**Table S3:** Results of the ROI analysis of cortical thickness.
**Table S4:** Results of the ROI analysis of cerebral blood flow.
**Table S5:** Significant correlations between neuropsychological testing scores and MRI metrics.

## Data Availability

The data that support the findings of this study are available from the corresponding author upon reasonable request.
