# Supplementary material for: Myo‐Inositol Deficiency, Structural Brain Changes, and Cerebral Perfusion Alterations in Classic Galactosemia: Preliminary Insights From a Multiparametric MRI Study
Source: J Inherit Metab Dis. 2025 Oct 13;48(6):e70097. doi: 10.1002/jimd.70097 (PMC12518697; doi:10.1002/jimd.70097)
Supplement: Supplementary file 1 — Table S1: Minimum Reporting Standards for in vivo MR Spectroscopy (MRSinMRS). Table S2: Results of the ROI analysis of cortical surface area. Table S3: Results of the ROI analysis of cortical thickness. Table S4: Results of the ROI analysis of cerebral blood flow. Table S5: Significant correlations between neuropsychological testing scores and MRI metrics. [file JIMD-48-0-s001.docx]

**SUPPLEMENTARY MATERIAL**

**Supplementary Table 1:** Minimum Reporting Standards for *in vivo* MR Spectroscopy (MRSinMRS)

| **Minimum Reporting Standards in MR Spectroscopy checklist (according to Lin et al. NMR Biomed 2021)** | | |
| --- | --- | --- |
| **1. Hardware** |  | |
| **a. Field strength [T]** | 7 | |
| **b. Manufacturer** | Siemens | |
| **c. Model (software version if available)** | Magnetom.plus 7T | |
| **d. RF coils: nuclei (transmit/ receive), number of channels, type, body part** | ^1^H (1Tx/32Rx), head, Nova Medical | |
| **e. Additional hardware** | N/A | |
| **2. Acquisition** |  |  |
| **a. Pulse sequence** | **3D-CRT-MRSI** | **semi-LASER SVS** |
| **b. Volume of Interest (VOI) locations** | Parallel to the anterior commissure-posterior commissure line, covering cerebrum from vertex to inferior to lateral ventricles | 1. Left putamen 2. Cerebellar vermis |
| **c. Nominal VOI size [cm^3^, mm^3^]** | 220×220×47 mm^3^ | 1. 35 × 10 × 15 mm³ 2. 25 × 10 × 25 mm³ |
| **d. Repetition Time (TR), Echo Time (TE) [ms, s]** | TR=320 ms / 1.3 ms acquisition delay | TR=8,600 ms / TE=28 ms |
| **e. Total number of Excitations or acquisitions per spectrum** | 1 average | 1. 64 averages 2. 71 averages |
| **f. Additional sequence parameters (spectral width in Hz, number of spectral points, frequency offsets); If STEAM: Mixing Time TM; If MRSI: 2D or 3D, FOV in all directions, matrix size, acceleration factors** | Bandwidth: 2,778 Hz, 558 spectral points, MRSI: 3D, FOV 220×220×133 mm^3^, matrix size 44×44×31 | Bandwidth: 6,000 Hz, 2,048 spectral points |
| **g. Water Suppression Method** | WET | VAPOR |
| **h. Shimming Method, reference peak, and thresholds for “acceptance of shim” chosen** | Standard shim + manual adjustment, water peak < 45 Hz | FASTMAP shimming, water peak < 30 Hz |
| **i. Triggering or motion correction method (respiratory, peripheral, cardiac triggering, incl. device used and delays)** | N/A | |
| **3. Data analysis methods and outputs** |  |  |
| **a. Analysis software** | LCModel 6.3-1 | |
| **b. Processing steps deviating from quoted reference or product** | N/A | |
| **c. Output measure (e.g. absolute concentration, institutional units, ratio)** | ratio | absolute concentration |
| **d. Quantification references and assumptions, fitting model assumptions** | Basis set simulated in NMRScope-B, macromolecular background | |
| **4. Data Quality** |  |  |
| **a. Reported variables (SNR, Linewidth (with reference peaks))** | SNR, FWHM (output of LCModel) | |
| **b. Data exclusion criteria** | CRLBs >20% | |
| **c. Quality measures of postprocessing Model fitting (e.g. CRLB, goodness of fit, SD of residual)** | CRLB | |
| **d. Sample Spectrum** | See Fig. 2 | See Fig. 3 |

**Supplementary Table 2:** Results of the ROI analysis of cortical surface area

|  | F | df | *p*_corr_ | η_p_^2^ | eMD [mm^2^] | SE |
| --- | --- | --- | --- | --- | --- | --- |
| *Left hemisphere* |  |  |  |  |  |  |
| LH caudal anterior cingulate | 1.366 | 1 | 0.172 | 0.121 | -182.1 | 59.4 |
| LH caudal middle frontal | 7.212 | 1 | 0.069 | 0.445 | -529.4 | 197.1 |
| LH cuneus | 0.497 | 1 | 0.541 | 0.051 | -117.3 | 169.5 |
| LH enthorinal | 7.237 | 1 | 0.069 | 0.446 | -113.1 | 42.1 |
| LH fusiform | 11.179 | 1 | 0.057 | 0.554 | -695.1 | 207.9 |
| LH inferior parietal | 2.641 | 1 | 0.172 | 0.227 | -395.7 | 243.5 |
| LH inferior temporal | 8.419 | 1 | 0.062 | 0.483 | -723.9 | 249.5 |
| LH isthmus cingulate | 6.564 | 1 | 0.069 | 0.422 | -178.6 | 69.7 |
| LH lateral occipital | 4.419 | 1 | 0.100 | 0.329 | -784.0 | 373.0 |
| LH lateral orbitofrontal | 14.098 | 1 | 0.052 | 0.610 | -715.2 | 190.5 |
| LH lingual | 3.217 | 1 | 0.137 | 0.263 | -621.4 | 346.4 |
| LH medial orbitofrontal | 6.987 | 1 | 0.069 | 0.437 | -213.7 | 80.9 |
| LH middle temporal | 1.100 | 1 | 0.357 | 0.109 | -284.3 | 271.1 |
| LH parahippocampal | 6.144 | 1 | 0.072 | 0.406 | -160.9 | 64.9 |
| LH paracentral | 5.329 | 1 | 0.084 | 0.372 | -308.1 | 133.5 |
| LH pars opercularis | 4.510 | 1 | 0.100 | 0.334 | -304.4 | 143.3 |
| LH pars orbitalis | 9.194 | 1 | 0.062 | 0.505 | -116.7 | 38.5 |
| LH pars triangularis | 6.597 | 1 | 0.069 | 0.423 | -380.2 | 148.0 |
| LH pericalcarine | 0.306 | 1 | 0.614 | 0.033 | -117.1 | 211.7 |
| LH postcentral | 0 | 1 | 0.993 | 0 | -1.0 | 205.9 |
| LH posterior cingulate | 33.506 | 1 | 0.057 | 0.788 | -406.8 | 70.3 |
| LH precentral | 1.405 | 1 | 0.305 | 0.135 | -409.5 | 345.5 |
| LH precuneus | 4.283 | 1 | 0.100 | 0.322 | -533.9 | 258.0 |
| LH rostral anterior cingulate | 10.042 | 1 | 0.057 | 0.527 | -326.8 | 103.1 |
| LH rostral middle frontal | 5.587 | 1 | 0.081 | 0.383 | -826.9 | 349.9 |
| **LH superior frontal** | **10.314** | **1** | **0.016** | **0.534** | **-1,644.2** | **512.0** |
| LH superior parietal | 3.758 | 1 | 0.115 | 0.295 | -856.6 | 441.9 |
| LH superior temporal | 2.08 | 1 | 0.218 | 0.188 | -669.3 | 464.1 |
| LH supramarginal | 8.643 | 1 | 0.062 | 0.490 | -743.0 | 252.7 |
| LH transverse temporal | 4.12 | 1 | 0.103 | 0.314 | 405.1 | 27.6 |
| LH insula | 4.831 | 1 | 0.096 | 0.349 | -194.3 | 88.4 |
| **LH mean surface area** | **16.358** | **1** | **0.003** | **0.645** | **-13,838.4** | **3,421.6** |
| *Right hemisphere* |  |  |  |  |  |  |
| RH caudal anterior cingulate | 0.910 | 1 | 0.419 | 0.092 | -117.1 | 122.8 |
| RH caudal middle frontal | 3.116 | 1 | 0.191 | 0.257 | -445.2 | 252.2 |
| RH cuneus | 5.493 | 1 | 0.124 | 0.379 | -340.8 | 145.4 |
| RH enthorinal | 3.428 | 1 | 0.188 | 0.276 | -71.8 | 38.8 |
| RH fusiform | 11.136 | 1 | 0.056 | 0.608 | -718.6 | 185.2 |
| RH inferior parietal | 5.572 | 1 | 0.124 | 0.382 | -842.4 | 356.9 |
| RH inferior temporal | 2.941 | 1 | 0.194 | 0.246 | -410.8 | 239.5 |
| RH isthmus cingulate | 10.502 | 1 | 0.058 | 0.539 | -299.1 | 92.3 |
| RH lateral occipital | 2.291 | 1 | 0.221 | 0.203 | -623.2 | 411.7 |
| RH lateral orbitofrontal | 8.583 | 1 | 0.066 | 0.488 | -741.8 | 253.2 |
| RH lingual | 3.628 | 1 | 0.184 | 0.287 | -560.1 | 294.1 |
| **RH medial orbitofrontal** | **22.902** | **1** | **0.016** | **0.718** | **-225.2** | **47.1** |
| RH middle temporal | 9.709 | 1 | 0.058 | 0.519 | -607.3 | 194.9 |
| RH parahippocampal | 4.830 | 1 | 0.134 | 0.349 | -71.9 | 32.7 |
| RH paracentral | 2.522 | 1 | 0.207 | 0.219 | -218.7 | 137.7 |
| RH pars opercularis | 2.738 | 1 | 0.195 | 0.233 | -208.8 | 126.2 |
| RH pars orbitalis | 0.742 | 1 | 0.439 | 0.076 | -70.9 | 82.3 |
| RH pars triangularis | 3.169 | 1 | 0.191 | 0.26 | -179.5 | 100.8 |
| RH pericalcarine | 0.808 | 1 | 0.434 | 0.082 | -186.9 | 207.9 |
| RH postcentral | 1.069 | 1 | 0.391 | 0.106 | -322.1 | 311.5 |
| RH posterior cingulate | 4.329 | 1 | 0.148 | 0.325 | -239.2 | 115.0 |
| RH precentral | 1.158 | 1 | 0.384 | 0.114 | -290.5 | 270.0 |
| RH precuneus | 5.446 | 1 | 0.124 | 0.377 | -621.3 | 266.3 |
| RH rostral anterior cingulate | 4.977 | 1 | 0.134 | 0.356 | -173.5 | 77.8 |
| **RH rostral middle frontal** | **19.084** | **1** | **0.016** | **0.680** | **-1,134.0** | **259.6** |
| **RH superior frontal** | **25.999** | **1** | **0.016** | **0.743** | **-1,985.3** | **389.4** |
| RH superior parietal | 9.486 | 1 | 0.058 | 0.513 | -900.0 | 292.2 |
| RH superior temporal | 0.365 | 1 | 0.580 | 0.039 | -205.3 | 340.0 |
| RH supramarginal | 0.188 | 1 | 0.675 | 0.02 | -105.1 | 242.3 |
| RH transverse temporal | 2.858 | 1 | 0.194 | 0.241 | -41.6 | 24.6 |
| RH insula | 1.162 | 1 | 0.384 | 0.114 | -133.2 | 123.6 |
| **RH mean surface area** | **14.137** | **1** | **0.004** | **0.611** | **-13,160.5** | **3,500.2** |

Notes: *p*_corr_: false discovery rate (FDR)-adjusted *p*; eMD: estimated mean difference; SE: standard error; η_p_^2^: partial eta-squared (= estimate of effect size); LH: left hemisphere; RH: right hemisphere

**Supplementary Table 3:** Results of the ROI analysis of cortical thickness

|  | F | df | *p*_corr_ | η_p_^2^ | eMD [mm] | SE |
| --- | --- | --- | --- | --- | --- | --- |
| *Left hemisphere* |  |  |  |  |  |  |
| LH caudal anterior cingulate | 9.531 | 1 | 0.055 | 0.514 | 0.213 | 0.069 |
| **LH caudal middle frontal** | **21.324** | **1** | **0.016** | **0.703** | **0.271** | **0.059** |
| LH cuneus | 8.838 | 1 | 0.055 | 0.495 | 0.217 | 0.073 |
| LH enthorinal | 1.926 | 1 | 0.247 | 0.176 | -0.15 | 0.108 |
| LH fusiform | 6.433 | 1 | 0.083 | 0.417 | 0.132 | 0.052 |
| LH inferior parietal | 0.948 | 1 | 0.394 | 0.095 | 0.128 | 0.132 |
| LH inferior temporal | 2.528 | 1 | 0.196 | 0.219 | 0.138 | 0.087 |
| LH isthmus cingulate | 4.21 | 1 | 0.114 | 0.319 | 0.242 | 0.118 |
| LH lateral occipital | 3.398 | 1 | 0.145 | 0.274 | 0.174 | 0.094 |
| LH lateral orbitofrontal | 2.453 | 1 | 0.196 | 0.214 | 0.176 | 0.113 |
| LH lingual | 8.049 | 1 | 0.059 | 0.472 | 0.278 | 0.098 |
| LH medial orbitofrontal | 4.45 | 1 | 0.114 | 0.331 | 0.296 | 0.14 |
| LH middle temporal | 0.088 | 1 | 0.774 | 0.01 | -0.035 | 0.118 |
| LH parahippocampal | 0.203 | 1 | 0.685 | 0.022 | 0.069 | 0.154 |
| LH paracentral | 6.769 | 1 | 0.082 | 0.429 | 0.257 | 0.099 |
| **LH pars opercularis** | **15.056** | **1** | **0.025** | **0.626** | **0.238** | **0.061** |
| LH pars orbitalis | 1.616 | 1 | 0.281 | 0.152 | 0.142 | 0.112 |
| LH pars triangularis | 5.64 | 1 | 0.091 | 0.385 | 0.235 | 0.099 |
| LH pericalcarine | 4.266 | 1 | 0.114 | 0.322 | 0.211 | 0.102 |
| LH postcentral | 2.669 | 1 | 0.193 | 0.229 | 0.15 | 0.092 |
| LH posterior cingulate | 5.47 | 1 | 0.091 | 0.378 | 0.142 | 0.061 |
| LH precentral | 0.444 | 1 | 0.558 | 0.047 | 0.065 | 0.097 |
| LH precuneus | 4.74 | 1 | 0.110 | 0.345 | 0.185 | 0.085 |
| **LH rostral anterior cingulate** | **19.272** | **1** | **0.021** | **0.682** | **0.535** | **0.082** |
| LH rostral middle frontal | 9.025 | 1 | 0.055 | 0.501 | 0.281 | 0.094 |
| **LH superior frontal** | **31.092** | **1** | **<0.001** | **0.776** | **0.337** | **0.061** |
| LH superior parietal | 3.488 | 1 | 0.145 | 0.279 | 0.122 | 0.065 |
| LH superior temporal | 1.68 | 1 | 0.318 | 0.157 | 0.127 | 0.098 |
| LH supramarginal | 5.637 | 1 | 0.091 | 0.385 | 0.184 | 0.077 |
| **LH transverse temporal** | **11.821** | **1** | **0.036** | **0.568** | **0.482** | **0.14** |
| **LH insula** | **14.833** | **1** | **0.025** | **0.493** | **0.397** | **0.103** |
| **LH mean thickness** | **11.37** | **1** | **0.008** | **0.558** | **0.175** | **0.052** |
| *Right hemisphere* |  |  |  |  |  |  |
| RH caudal anterior cingulate | 1.334 | 1 | 0.345 | 0.129 | 0.115 | 0.100 |
| **RH caudal middle frontal** | **27.807** | **1** | **<0.001** | **0.755** | **0.347** | **0.066** |
| RH cuneus | 6.602 | 1 | 0.072 | 0.423 | 0.222 | 0.086 |
| RH enthorinal | 9.443 | 1 | 0.053 | 0.512 | -0.432 | 0.141 |
| RH fusiform | 6.323 | 1 | 0.073 | 0.413 | 0.139 | 0.055 |
| RH inferior parietal | 3.279 | 1 | 0.140 | 0.267 | 0.117 | 0.065 |
| RH inferior temporal | 0.004 | 1 | 0.952 | 0.000 | 0.003 | 0.053 |
| RH isthmus cingulate | 5.793 | 1 | 0.081 | 0.392 | 0.343 | 0.142 |
| RH lateral occipital | 4.640 | 1 | 0.090 | 0.340 | 0.111 | 0.052 |
| RH lateral orbitofrontal | 8.099 | 1 | 0.054 | 0.474 | 0.396 | 0.139 |
| RH lingual | 4.762 | 1 | 0.090 | 0.346 | 0.174 | 0.080 |
| **RH medial orbitofrontal** | **26.145** | **1** | **<0.001** | **0.744** | **0.483** | **0.095** |
| RH middle temporal | 0.383 | 1 | 0.590 | 0.041 | 0.073 | 0.117 |
| RH parahippocampal | 0.295 | 1 | 0.620 | 0.032 | 0.074 | 0.136 |
| **RH paracentral** | **23.564** | **1** | **<0.001** | **0.724** | **0.205** | **0.042** |
| RH pars opercularis | 6.972 | 1 | 0.070 | 0.437 | 0.213 | 0.081 |
| RH pars orbitalis | 0.949 | 1 | 0.423 | 0.095 | 0.117 | 0.120 |
| RH pars triangularis | 4.782 | 1 | 0.090 | 0.347 | 0.210 | 0.096 |
| RH pericalcarine | 4.800 | 1 | 0.090 | 0.348 | 0.241 | 0.110 |
| RH postcentral | 3.316 | 1 | 0.140 | 0.269 | 0.163 | 0.090 |
| RH posterior cingulate | 9.177 | 1 | 0.053 | 0.505 | 0.196 | 0.065 |
| RH precentral | 2.103 | 1 | 0.234 | 0.189 | 0.088 | 0.061 |
| RH precuneus | 8.601 | 1 | 0.053 | 0.489 | 0.164 | 0.056 |
| **RH rostral anterior cingulate** | **14.873** | **1** | **0.021** | **0.623** | **0.458** | **0.119** |
| **RH rostral middle frontal** | **17.999** | **1** | **0.012** | **0.667** | **0.353** | **0.083** |
| **RH superior frontal** | **56.922** | **1** | **<0.001** | **0.863** | **0.371** | **0.049** |
| RH superior parietal | 4.580 | 1 | 0.090 | 0.337 | 0.082 | 0.038 |
| RH superior temporal | 0.505 | 1 | 0.548 | 0.053 | 0.087 | 0.123 |
| RH supramarginal | 0.658 | 1 | 0.503 | 0.068 | 0.061 | 0.075 |
| RH transverse temporal | 4.954 | 1 | 0.090 | 0.355 | 0.298 | 0.134 |
| RH insula | 8.743 | 1 | 0.053 | 0.493 | 0.307 | 0.104 |
| **RH mean thickness** | **17.444** | **1** | **0.002** | **0.660** | **0.176** | **0.042** |

Notes: *p*_corr_: false discovery rate (FDR)-adjusted *p*; eMD: estimated mean difference; SE: standard error; ηp^2^: partial eta-squared (= estimate of effect size); LH: left hemisphere; RH: right hemisphere

**Supplementary Table 4:** Results of the ROI analysis of cerebral blood flow

|  | t | df | *p*_corr_ | MD [ml/100g/min] | SE |
| --- | --- | --- | --- | --- | --- |
| *Left hemisphere* |  |  |  |  |  |
| Left cerebellum WM | 0.682 | 9 | 0.512 | 4.71 | 6.91 |
| Left cerebellum cortex | 0.293 | 9 | 0.776 | 1.64 | 5.58 |
| **Left thalamus** | **2.445** | **9** | **0.037** | **14.67** | **6.00** |
| Left putamen | 0.996 | 9 | 0.345 | 5.24 | 5.26 |
| Left caudate | 0.715 | 9 | 0.493 | 4.56 | 6.38 |
| Left pallidum | 1.394 | 9 | 0.197 | 3.62 | 2.60 |
| Left hippocampus | 2.177 | 9 | 0.057 | 8.69 | 3.99 |
| **Left amygdala** | **2.760** | **9** | **0.022** | **7.55** | **2.74** |
| LH caudal anterior cingulate | 0.521 | 9 | 0.615 | 1.86 | 3.57 |
| LH caudal middle frontal | 1.333 | 9 | 0.215 | 9.13 | 6.85 |
| LH cuneus | 0.451 | 9 | 0.663 | 2.86 | 6.35 |
| LH fusiform | 0.910 | 9 | 0.386 | 3.83 | 4.21 |
| LH inferior parietal | 1.493 | 9 | 0.170 | 8.69 | 5.82 |
| LH inferior temporal | 1.439 | 9 | 0.184 | 6.48 | 4.51 |
| LH isthmus cingulate | 1.032 | 9 | 0.329 | 4.36 | 4.22 |
| LH lateral occipital | 0.966 | 9 | 0.359 | 6.46 | 6.68 |
| LH lateral orbitofrontal | 0.575 | 9 | 0.579 | 2.67 | 4.62 |
| LH lingual | 0.572 | 9 | 0.581 | 3.73 | 6.51 |
| LH medial orbitofrontal | 0.808 | 9 | 0.440 | 2.99 | 3.70 |
| LH middle temporal | 1.318 | 9 | 0.220 | 6.48 | 4.91 |
| LH parahippocampal | 1.964 | 9 | 0.081 | 6.48 | 3.30 |
| LH paracentral | 1.200 | 9 | 0.261 | 5.72 | 4.77 |
| LH pars opercularis | 1.961 | 9 | 0.081 | 9.08 | 4.63 |
| LH pars orbitalis | -0.132 | 9 | 0.898 | -0.81 | 6.13 |
| LH pars triangularis | 2.092 | 9 | 0.079 | 9.87 | 4.94 |
| LH pericalcarine | 0.244 | 9 | 0.813 | 1.39 | 5.69 |
| LH postcentral | 1.381 | 9 | 0.200 | 6.39 | 4.63 |
| LH posterior cingulate | 1.636 | 9 | 0.136 | 6.87 | 4.20 |
| LH precentral | 1.570 | 9 | 0.151 | 7.53 | 4.80 |
| LH precuneus | 1.511 | 9 | 0.165 | 7.17 | 4.74 |
| LH rostral anterior cingulate | 1.907 | 9 | 0.089 | 5.47 | 2.87 |
| LH rostral middle frontal | 1.814 | 9 | 0.103 | 10.66 | 5.88 |
| LH superior frontal | 1.551 | 9 | 0.155 | 8.68 | 5.60 |
| LH superior parietal | 1.203 | 9 | 0.260 | 7.03 | 5.84 |
| **LH superior temporal** | **2.302** | **9** | **0.047** | **7.77** | **3.37** |
| LH supramarginal | 1.172 | 9 | 0.271 | 5.93 | 5.06 |
| **LH transverse temporal** | **2.463** | **9** | **0.036** | **5.94** | **2.41** |
| LH insular | 2.118 | 9 | 0.063 | 6.44 | 3.04 |
| *Right hemisphere* |  |  |  |  |  |
| Right cerebellum WM | 0.522 | 9 | 0.615 | 3.53 | 6.77 |
| Right cerebellum cortex | 0.396 | 9 | 0.701 | 2.29 | 5.77 |
| **Right thalamus** | **2.433** | **9** | **0.038** | **13.03** | **5.36** |
| Right putamen | 1.848 | 9 | 0.098 | 9.56 | 5.18 |
| Right caudate | 0.747 | 9 | 0.474 | 4.64 | 6.20 |
| **Right pallidum** | **2.774** | **9** | **0.022** | **9.53** | **3.43** |
| **Right hippocampus** | **3.071** | **9** | **0.013** | **10.95** | **3.57** |
| **Right amygdala** | **3.193** | **9** | **0.011** | **14.12** | **4.42** |
| RH caudal anterior cingulate | 1.051 | 9 | 0.321 | 3.77 | 3.56 |
| RH caudal middle frontal | 1.298 | 9 | 0.226 | 9.21 | 7.09 |
| RH cuneus | 0.840 | 9 | 0.423 | 5.43 | 6.46 |
| RH fusiform | 1.231 | 9 | 0.250 | 4.76 | 3.87 |
| RH inferior parietal | 1.193 | 9 | 0.264 | 7.30 | 6.12 |
| RH inferior temporal | 1.315 | 9 | 0.221 | 8.59 | 6.53 |
| RH isthmus cingulate | 0.892 | 9 | 0.396 | 5.04 | 5.66 |
| RH lateral occipital | 1.115 | 9 | 0.294 | 7.66 | 6.87 |
| RH lateral orbitofrontal | 0.363 | 9 | 0.725 | 1.64 | 4.53 |
| RH lingual | 1.027 | 9 | 0.331 | 5.78 | 5.63 |
| RH medial orbitofrontal | 0.643 | 9 | 0.537 | 2.02 | 3.15 |
| RH middle temporal | 1.369 | 9 | 0.204 | 7.71 | 5.63 |
| **RH parahippocampal** | **2.275** | **9** | **0.049** | **8.44** | **3.71** |
| RH paracentral | 0.858 | 9 | 0.413 | 4.29 | 5.00 |
| RH pars opercularis | 2.021 | 9 | 0.074 | 10.50 | 5.19 |
| RH pars orbitalis | 0.725 | 9 | 0.487 | 5.08 | 7.01 |
| RH pars triangularis | 1.112 | 9 | 0.295 | 6.08 | 5.47 |
| RH pericalcarine | 1.032 | 9 | 0.329 | 6.26 | 6.07 |
| RH postcentral | 1.162 | 9 | 0.275 | 6.34 | 5.46 |
| RH posterior cingulate | 1.376 | 9 | 0.202 | 5.71 | 4.15 |
| RH precentral | 1.355 | 9 | 0.104 | 7.02 | 5.18 |
| RH precuneus | 1.301 | 9 | 0.225 | 6.86 | 5.27 |
| **RH rostral anterior cingulate** | **2.471** | **9** | **0.036** | **7.72** | **3.13** |
| RH rostral middle frontal | 1.807 | 9 | 0.104 | 11.85 | 6.56 |
| RH superior frontal | 1.473 | 9 | 0.175 | 8.35 | 5.67 |
| RH superior parietal | 1.556 | 9 | 0.154 | 8.50 | 5.46 |
| RH superior temporal | 2.250 | 9 | 0.051 | 8.00 | 3.56 |
| RH supramarginal | 1.513 | 9 | 0.165 | 7.94 | 5.25 |
| RH transverse temporal | 1.308 | 9 | 0.223 | 5.97 | 4.57 |
| RH insular | 2.179 | 9 | 0.057 | 7.51 | 3.45 |

Notes: *p*_corr_: false discovery rate (FDR)-adjusted *p*; MD: mean difference; SE: standard error; LH: left hemisphere; RH: right hemisphere

| Neuropsychological test | Brain measure | *r* value | *p* value |
| --- | --- | --- | --- |
| Beery Visual-Motor Integration (VMI) Test | *Volumetry* |  |  |
|  | Left cerebellum white matter | 0.999 | 0.033 |
|  | Left cerebellum cortex | 0.996 | 0.046 |
|  | Right cerebellum cortex | 0.999 | 0.028 |
| Beery VMI Test | *Cortical thickness* |  |  |
|  | Right pericalcarine (V1) | -0.986 | 0.014 |
|  | Left pericalcarine (V1) | -0.952 | 0.048 |
|  | Left lateral occipital | -0.955 | 0.045 |
|  | Right fusiform | -0.951 | 0.049 |
|  | Right isthmus cingulate | -1 | 0.002 |
|  | Left isthmus cingulate | -0.999 | 0.034 |
| Verbal Learning Memory Test (VLMT) – Dg 1-5 | *MR spectroscopy* |  |  |
|  | Cerebellar Ins | 0.999 | 0.023 |
| VLMT: Recall after interference – Dg 6 | *Cortical thickness* |  |  |
|  | Right isthmus cingulate | -0.953 | 0.047 |
| VLMT: Delayed recall – Dg 7 | *Cortical thickness* |  |  |
|  | Right isthmus cingulate | -0.963 | 0.037 |
| Test of Attentional Performance (TAP): intrinsic alertness* | *Cortical thickness* |  |  |
|  | Right rostral middle frontal | 1 | 0.012 |
|  | Right precentral | 0.995 | 0.044 |
|  | Right superior parietal | 0.997 | 0.045 |
| TAP: cognitive flexibility* | *Cortical thickness* |  |  |
|  | Left rostral middle frontal | 1 | 0.002 |
|  | Left lateral occipital | 1 | 0.012 |
|  | Left middle temporal | 0.998 | 0.036 |

**Supplementary Table 5:** Significant correlations between neuropsychological testing scores and MRI metrics.

Notes: * TAP results were available only for three patients
